# Provenance and Funding of Extremely Cited Biomedical Articles Published Between 2003 and 2024

**DOI:** 10.1001/jamahealthforum.2025.3045

**Published:** 2025-09-12

**Authors:** John P.A. Ioannidis

**Affiliations:** 1 Department of Medicine, Stanford University, Stanford, California; 2 Department of Epidemiology and Population Health, Stanford University, Stanford, California; 3 Department of Biomedical Data Science, Stanford University, Stanford, California; 4 Meta-Research Innovation Center at Stanford (METRICS), Stanford University, Stanford, California

## Abstract

**Importance:**

It is important to monitor changes in the biomedical literature and its funding. China has surpassed the US in publications and, in some analyses, in some impact indicators.

**Objective:**

To evaluate changes over time in the profiles of the most highly cited biomedical publications.

**Design, Setting, and Participants:**

This cross-sectional study assessed 100 top-cited biomedical articles (based on Scopus) published in each of 3 time periods (2003-2004, 2013-2014, and 2023-2024) for corresponding authors, types of publications represented, and funding sources, with an emphasis on funding from the US National Institutes of Health (NIH), which has been traditionally considered the major funder of biomedical research.

**Main Outcomes and Measures:**

The main outcomes of interest are descriptive and include provenance (country of corresponding author), type of publication, type of funding, overall NIH funding, and NIH funding as the sole funding source.

**Results:**

Among 100 top-cited biomedical publications, corresponding authors from the US decreased over time (59 of 100 articles in 2003-2004, 58 of 100 in 2013-2014, 45 of 100 in 2023-2024). Corresponding authors from China represented 0 top-cited publications in 2003-2004, 1 in 2013-2014, and 4 in 2023-2024. There was an increase in consensus articles (10 in 2003-2004 vs 24 in 2023-2024) and in reference statistics articles (1 in 2003-2004, 10 in 2013-2014, and 11 in 2023-2024). Reviews remained common among top-cited articles, but almost always were nonsystematic. NIH funding was listed in 45 publications in 2003-2004, 50 in 2013-2014, and 23 in 2023-2024. All other countries combined surpassed US public funding in 2023-2024. Funding by NIH in combination with other funders increased from 13 articles in 2003-2204 to 22 and 21, respectively, in 2013-2014 and 2023-2024, but funding by NIH alone decreased in the last decade (32 of 100 in 2002-2003, 28 of 100 in 2013-2014, and 2 of 100 in 2023-2024). More commonly listed funding from nonprofit organizations, societies, and institutions complemented the NIH funding decline. The first authors of 7 of 45 and the corresponding authors of 14 of 45 top-cited US-based articles of 2023-2024 were listed as leaders of active NIH grants in RePORTER as of February 2025. Citation gaming became more obvious in 2023-2024 with the advent of some atypical highly cited papers and a larger share of nonsystematic reviews and consensus documents.

**Conclusions and Relevance:**

Results of this study suggest that, overall, the US remains a world leader regarding the most highly cited biomedical research and NIH funding retains a substantial presence among top-cited articles. However, NIH influence had decreased overall, and top-cited articles funded exclusively by NIH have almost disappeared. Strengthening public funding is essential to secure research that serves the common good.

## Key Points

**Question:**

Has the US, and the National Institutes of Health (NIH) in particular, lost their dominance in supporting the most highly cited biomedical articles?

**Findings:**

In this cross-sectional study of the 100 top-cited biomedical publications in 2003-2004, 2013-2014, and 2023-2024, the US remained dominant, with a small decrease in corresponding authors of the top-cited publications (59 of 100 in 2003-2004, 58 of 100 in 2013-2014, and 45 of 100 in 2023-2024). Involvement of NIH funding decreased in 2023-2024, and in 2023-2024 only 2 of 100 articles were funded exclusively by the NIH.

**Meaning:**

These findings suggest that as of 2024 the US remained a world leader in top-cited biomedical research, but the influence of NIH funding had decreased and influential work depended on funding from diverse funders.

## Teaser

This cross-sectional study explores the origin and funding of top-cited biomedical publications with regard to numbers of articles originating in the US and supported by the National Institutes of Health.

## Introduction

The landscape of biomedical publications is changing over time in volume and provenance. The US has long led in the number of overall publications and high-impact work. However, China now ranks first in numbers of annual publications, spends more on research and development (R&D), and has more researchers.^1,2,3^ Some analyses suggest that, in 2022, China outperformed the US even in the number of articles published in 82 high-quality journals,^4^ and also excelled in numbers of articles in the top 1% of citations.^5^ Concurrently, major changes and challenges have emerged in research funding and the publication ecosystem. The US National Institutes of Health (NIH) has traditionally been the primary biomedical research funder but is currently threatened with major cuts.^6^ Investigators have struggled to secure funding from diverse sources. Commercial publishers have large profit margins from a burgeoning publication market.^7,8^ This market is subject to the inflation and manipulation (gaming) of journal impact factors.^9^ Journals may also try to publish more articles that concentrate extremely high citations.^10^ Many types of gaming practices abound.^11,12^

The current analysis aims to examine the most extremely cited biomedical articles published in 2003-2004, 2013-2014, and 2023-2024. The following questions are asked: what is the provenance of these articles and what types of articles reached the extremes of citation distributions? Has the US indeed decreased and has China overtaken the US in citation extremes? What are the funding sources of extremely cited articles? Has the NIH continued to be the major funder of that work? Finally, might some extremely cited articles reflect citation gaming?

## Methods

### Search Strategy and Eligible Articles

The analysis used Scopus citation data.^13^ Citations have limitations, but they do carry weight and influence in appraising scientific work, and they are also objective reproducible measurements.

The analysis is exploratory, and no protocol was preregistered. No institutional review board approval was requested as this was a bibliometric analysis using public bibliometric data, and there is also no reporting guideline for such articles.

Only major articles were considered (Scopus categories: articles, reviews, and conference papers). Two periods were considered initially: major articles published in 2003-2004 and those published in 2023-2024. However, with differences observed, a third cohort for 2013-2014 was added to probe whether changes were gradual. Searches for the initial 2 cohorts were performed on January 24, 2025, and for the 2013-2014 cohort 1 month later. For each calendar period, all articles were ordered in decreasing number of citations received. Starting from the most-cited, publications were screened to identify those relevant to biomedicine and the top 100 most highly cited biomedical publications were retrieved for each of the time periods. Publications were deemed relevant to biomedicine if they belonged to any of the following Scopus subject areas: medicine; biochemistry, genetics, and molecular biology; pharmacology, toxicology, and pharmaceutics; immunology and microbiology; neuroscience; nursing; health professions; veterinary; and dentistry. Articles cataloged in other subject areas by Scopus could be included only if they were deemed to have major primary biomedical interest.

### Data Extraction and Definitions

For the 3 batches of eligible articles, meta-data were exported from Scopus on bibliographic information, citations, funding information, and corresponding authors and their addresses. Moreover, full texts were downloaded to verify Scopus-extracted information and to complement missing information not captured by Scopus.

Country of origin was assigned based on the corresponding authors. Eligible publications were classified as primary research: resources and methods (including new software, databases, methods [statistical, computational, or other] and tools); and other primary research: reviews; consensus articles (including guidelines, recommendations, consensus documents, and definitions); and reference statistics for diseases and conditions. Funding was classified as NIH, other US federal/public (eg, National Science Foundation, Department of Energy, Centers for Disease Control and Prevention), other government/public from other countries, nonprofit (eg, Gates Foundation or Wellcome Trust), for-profit (eg, pharmaceutical or tech companies), societies (eg, American Heart Association), and institutional (eg, universities). When funding was listed from multiple categories, all were captured. Articles with listed NIH funding were also categorized as listing only NIH funding or listing additional funders.

### Data Assessment

The main outcomes of interest were descriptive and include provenance (country of corresponding author), type of publication, type of funding, overall NIH funding, and NIH funding as the sole funding source. Furthermore, for the 2023-2024 cohort, the first authors and corresponding authors of articles with US corresponding authors were assessed in NIH RePORTER^14^ to see if they were listed as of February 2025 as leaders of any NIH grants. Finally, it was assessed whether any highly cited articles had atypical features, such as more than 20% of the citations derived from a single journal, journal subject matter and/or title not representing the content of the article, or retraction (per the Retraction Watch database^13^). This study’s aims were descriptive without testing hypotheses with statistical tests.

## Results

### Scientific Literature and Identification of Top-Cited Publications

In this cross-sectional study of 3 time periods over 2 decades, China was close to the US in productivity by 2013-2014 and had almost double the productivity of the US in 2023-2024 across the entire scientific literature; the difference was more modest for the biomedical literature (Table 1). For all time periods, biomedical articles accounted for most of the extremely cited articles across science. We had to screen 190 top-cited articles in 2003-2004, 185 in 2013-2014, and 162 in 2023-2024 to identify 100 biomedical publications.

**Table 1.** Top 5 Countries With Authors Among All Full Articles^a^

Country	Full articles across all sciences (in biomedicine), No.^b^		
	2003-2004	2013-2014	2023-2024
US	832 986 (366 183)	1 134 424 (497 623)	1 251 053 (582 986)
Japan	224 808 (96 225)	253 129 (100 324)	255 483 (107 933)^c^
UK	212 575 (96 639)	323 403 (134 116)	412 215 (165 511)
Germany	202 425 (83 166)	311 383 (114 978)	367 622 (134 321)
China	197 638 (49 099)^c^	928 110 (208 047)	2 234 689 (606 424)
India	65 802 (24 407)^c^	233 392 (82 173)^c^	573 549 (152 891)

^a^ A total of 2 921 896 full articles were indexed in Scopus for 2003-2004, while 5 032 647 were indexed for 2013-2014 and 7 550 657 were indexed for 2023-2024. Biomedical subject matter disciplines in Scopus include medicine; biochemistry, genetics and molecular biology; pharmacology, toxicology, and pharmaceutics; immunology and microbiology; neuroscience; nursing; health professions; veterinary; and dentistry. The total full articles in these subject matter Scopus categories were 1 181 567 in 2003-2004, 1 812 401 in 2013-2014, and 2 599 013 in 2023-2024 (notably, additional articles in other disciplines may also have some high relevance for biomedicine and these are not counted in these numbers). In 2003-2004 biomedical articles, France was ranked fifth with 57 414 full articles but was not among the top 5 in biomedical publications in any other time period or in total full articles in any time period.

^b^ Values in parentheses are biomedical publications based on Scopus subject matter disciplines.

^c^ Not ranked among the top 5 countries.

### Characteristics of Top-Cited Biomedical Publications

Details on the 100 most-cited biomedical articles published in each time period appear in eTables 1, 2, and 3 in Supplement 1 and Table 2. A majority of articles in the 2003-2004 cohort (59 of 100) and 2013-2014 cohort (58 of 100) had US corresponding authors, while in the 2023-2004 cohort only 44 of 100 articles had US corresponding authors. In the 2023-2024 cohort there were 7 other countries with corresponding authors in at least 4 articles (6 of 7 European) vs only 3 such countries in earlier cohorts. Eighteen publications had corresponding authors from multiple countries in the 2023-2024, while this was not seen in the 2003-2004 cohort and had occurred only twice in the 2013-2014 cohort. China had corresponding authors for 4 articles in 2023-2024, 1 article (in Hong Kong) in 2013-2014, and none in 2003-2004. In addition to China, 2 other non–high-income countries (India and Russia) also had 1 top-cited publication each in 2013-2014, and 5 other non–high-income countries had corresponding authors in at least 1 article in 2023-2024 (India, 2 articles; 1 article each for Colombia, Northern Cyprus, Jordan, and Malaysia). Conversely, all articles in 2003-2004 had corresponding authors from high-income countries.

**Table 2.** Characteristics of the 100 Most-Cited Biomedical Articles Published in 2003-2004, 2013-2014, and 2023-2024

Characteristic	Publication year for the most-cited articles, No.		
	2003-2004	2013-2014	2023-2024
Range of citations	4983-48 466	4403-53 866	430-9785
Corresponding author in US^a^	59	58	45
Other countries with corresponding authors in ≥4 articles			
United Kingdom	8	10	16
Germany	5	8	6
Canada	4	NQ	NQ
Spain	NQ	NQ	6
France	NQ	5	5
Italy	NQ	NQ	4
Switzerland	NQ	NQ	4
China	NQ	NQ	4
Corresponding authors in non–high-income countries	0	3	10
Type of publication			
Primary research^b^	66	55	37
Resources and methods	34	30	17
Other	32	25	20
Reviews	23	20	28
Nonsystematic	22	19	26
Systematic	1	1	2
Consensus articles^c^	10	15	24
Reference statistics^c^	1	10	11

Abbreviation: NQ, not qualifying (fewer than 4 articles).

^a^ A few society-based articles did not explicitly list corresponding authors but were then allocated to the country of the society (unless the society was not based in a single country).

^b^ Some methodological articles are difficult to classify in the subgroup resources and methods vs other; only 1 of the 2 was chosen to avoid double counting.

^c^ Consensus articles and reference statistics may sometimes also use review methods (including systematic review methods), but they are not classified as reviews so as to keep the categories of types of article mutually exclusive.

In 2003-2004, two-thirds of articles (66 of 100) were primary research publications, which were equally divided between resources and methods work and other primary research. There were 23 reviews, of which only one aimed to be systematic; 10 consensus items; and 1 reference statistics publication. The representation of primary research articles decreased gradually over time (55 of 100 in 2013-2014 and 37 of 100 in 2023-2024), relatively equally divided between resources and methods and other work. Reviews retained a considerable proportion of the publications, with almost all of them being nonsystematic. Over one-third of the top-cited articles in the 2023-2024 cohort were either consensus items (n = 24) or reference statistics (n = 11), with the increase in these types of articles seen even in the 2013-2014 cohort.

### Funding of Top-Cited Articles

The NIH was the leading single governmental funder in all 3 time-period cohorts, but the number of top-cited articles with any listed NIH funding decreased in the last decade and decreased by approximately half in the 2023-2024 cohort vs in the 2003-2004 cohort or the 2013-2014 cohort (23 vs 45 or 50, respectively) (Table 3). When limited to primary research, NIH was listed as a funder in 15 of 37 top-cited articles in the 2023-2024 cohort, 33 of 55 in the 2003-2004 cohort, and 32 of 66 in the 2013-2014 cohort.

**Table 3.** Funding for the 100 Most-Cited Biomedical Articles Published in 2003-2004, 2013-2014, and 2023-2024

Funding source	Publication year for the most-cited articles, No.		
	2003-2004	2013-2014	2023-2024
NIH	45	50	23
Other US federal/public	4	9	4
Other country public/government^a^	29	43	37
Nonprofit	13	21	24
For profit	14	8	22
Societies^b^	6	9	25
Institutional	4	11	18
None listed	21	7	16

Abbreviation: NIH, National Institutes of Health.

^a^ Includes international government-funded organizations such as the International Agency for Research on Cancer, World Health Organization, and United Nations.

^b^ When an article was generated by 1 or more societies, these were considered to have funded the effort, even if funding was not explicitly listed.

The sum for each cohort exceeds 100, because some articles listed more than 1 category of funding.

In 2023-2024, all other countries combined exceeded the NIH and other US federal agencies in top-cited articles. All European countries combined (corresponding authors in 42 of 100 articles) matched the NIH in 2023-2024, while China only funded 3 top-cited articles. In 2023-2024, there were large increases in the numbers of top-cited articles funded by nonprofit organizations and those listing institutional support, and even more so for publications supported by societies (Table 3). Some of this increase was seen already in 2013-2014 and was accentuated in the following decade.

In 2003-2004, a majority of NIH-funded publications (32 of 45 [71%]) did not list any other funders and this was similar in 2013-2014 (28 of 50 [56%]) (Table 4). Conversely, there were only 2 of 23 NIH-funded publications (9%) that did not list any other funders in the 2023-2024 cohort. Cofunding with nonprofit organizations was common in all cohorts, but in 2023-2024, there was also common cofunding with for-profit entities, public/government funding from other countries, and listed institutional support, which was less common in earlier time periods.

**Table 4.** Coexistence of Other Funders in the NIH-Funded Top-Cited Articles

Funder status	Publication year for the most-cited articles, No.		
	2003-2004 (n = 45)	2013-2014 (n = 50)	2023-2024 (n = 23)
By presence of other funders			
NIH alone	32	28	2
NIH and other listed funders	13	22	21
By type of other funders			
Other US federal/public	2	7	3
Other country public/government^a^	3	16	8
Nonprofit	8	11	13
For profit	4	5	7
Societies^b^	2	3	2
Institutional	2	4	7

Abbreviation: NIH, National Institutes of Health.

^a^ Includes international government-funded organizations such as the International Agency for Research on Cancer, World Health Organization, and United Nations.

^b^ When a publication was generated by 1 or more societies, these were considered to have funded the effort, even if funding was not explicitly listed.

### Current NIH Funding for First and Corresponding Authors

Of the 45 top-cited articles of 2023-2024 with US-based corresponding authors, in 7 (16%) the first author was listed in RePORTER as leading a currently active NIH grant. The same was observed for at least 1 of the corresponding authors in 14 articles (31%).

### Atypical Articles

One article in 2023^15^ was a guideline for reporting surgical case reports, and more than 98% of its citations had been made from articles published in the same journal, which publishes many surgical case reports. Another article in 2023^16^ was published in a chemistry journal, and the title suggested that it was providing cancer statistics and projections, but the content was a review of diverse cancers without substantive epidemiological projections. Apparently, the article was heavily cited by authors who mistook it for other regularly updated articles on cancer statistics. No top-cited publications in any time-period cohort had been retracted.

## Discussion

Biomedical research represents the majority of the most highly cited scientific literature and US-based authors continue to dominate the most influential scientific literature in biomedicine, despite a modest decline in the last decade. In contrast with previous work focused on publications in prestigious journals^4^ or the top 1% of citations,^5^ in which China apparently outpaced the US, the current analysis shows that China still had a negligible presence among the 100 most highly cited recent biomedical publications as of 2024. The NIH continues to be commonly listed as a funder. Nevertheless, in recent articles, the NIH is listed only approximately half as often compared with 1 or 2 decades ago. More prominently, in the recent cohort of extremely highly cited publications, the NIH is rarely listed as the only funder, in contrast with just a decade ago.

Nonprofit organizations, societies, and institutional support were increasingly listed as funders in top-cited publications. Recent articles tended to have a wider range of acknowledged funders. Perhaps reporting of funders has become more detailed, on average, over time. Investigators may take more care to acknowledge support and/or journals may be more explicit in funding disclosures.^17^ Some institutional support is likely to have been and continue to be common in all work from universities and research institutions. However, in the past, this has typically not been acknowledged explicitly. Public funding may also have been misclassified. Some investigators may not have listed their NIH funding, even if relevant to the work. Conversely, some other investigators may have listed NIH funding even if irrelevant to the published work. The latter pattern may become more frequent if investigators are increasingly pressured to demonstrate productivity to sponsors.

Increased collaboration, including international collaboration, can also be seen as a positive development. However, while other funders may cover some of the gap left by public research funding,^18^ public funding of the form provided by NIH is important to strengthen. Funding cuts may exacerbate the pressure to game the system. The large majority of the first authors or even the corresponding authors (who may be more senior, on average) of the most recent US-based extremely highly cited biomedical publications do not lead any currently active NIH grants. This is consistent with earlier analyses of NIH funding.^19^ More rather than less funding is therefore needed. Still, evidence-based efforts at research reform are welcome, including assessment of review, evaluation, and funding distribution methods.^20,21,22^

The top-cited articles of 2023-2024 show an increasing footprint of the pressure to boost impact metrics (eg, through publication of atypical papers that serve as handy-to-cite references, consensus documents, and nonsystematic reviews). The advent of massive publications has been associated with questionable incentives and even an increasing rate of fraud.^23,24^ This is particularly true for China.^25,26^ Outright fraud may be distinctly uncommon in the sample of extremely cited articles analyzed here, but gaming is likely; for example, the share of consensus items has increased markedly compared with earlier periods. Guidelines and other consensus documents have seen major increases in production.^27^ Consensus documents can be important, but the methodological rigor, and protection from conflicts for most of them, is questionable.^28,29^ Some journals have boosted their impact factors by engaging aggressively in the production of consensus documents. Another type of publication that achieves similar goals is the release of reference statistics. They can also be valuable, but they also provide routine citations for introduction sections. There is less methodological research on the validity of these exercises, but some early concerns have been raised even for the most rigorous among them.^30^

The popularity of such articles may also lead to the publication of articles that mimic their titles and thus share citation success. Authors may not read the articles they cite^31^ and titles may mislead about the content. Another atypical situation documented here is the publication of a reporting guidance article that is widely cited in articles appearing in the publishing journal. Finally, the considerable prevalence of reviews among the extremely cited publications is unsurprising. Reviews can attract many citations and often reflect the culmination or integration of important work and insightful syntheses. A worrisome pattern is the pervasive dearth of systematic approaches among extremely cited reviews. Several biases might have been curtailed with systematic methods.^32,33^ In some fields, especially in basic science, nonsystematic reviews still draw a lot of attention, and they may be thought to provide a broader overview of the field. However, higher adoption of systematic review methods would also improve basic science.^32^

Previous work has shown that articles listing NIH funding are, on average, more cited than other biomedical articles.^34^ According to PubMed with a simple search of NIH (Grants and Funding), the proportion of PubMed items listing NIH funding was 10.2% in 2003-2004 (125 906 of 1 234 380), 11.5% in 2013-2014 (270 533 of 2 355 828), and decreased to 7.4% in 2023-2024 (253 468 of 3 430 386). Therefore, the diminishing presence of the NIH is also seen in all articles not only the extremely highly cited ones. The NIH budget markedly increased between 1998 and 2004, then remained stagnant for a decade, and has increased by almost 50% in the last decade, although increases barely matched cumulative inflation.^35^ Increased funding phases may have translated to higher productivity. However, it takes substantial time lags for funded work to be conducted and published. A study of NIH-funded articles found an average of 8 publications per grant over 20 years.^36^

Beyond highly cited articles, other indicators may be examined to obtain a comprehensive view of the evolution and current state of the research ecosystem in the US, China, and globally. China has outpaced the US not only in the number of overall publications but also in patent applications (1 619 268 vs 594 340 in 2023), patent grants (798 347 vs 323 410 in 2023),^37^ and new business applications (24.8 million in the first 9 months of 2023^38^ vs 5.5 million during all of 2023^39^). US publications have a higher rate of international collaboration than China (40% vs 22% in 2023), but many other countries have even higher rates of international collaboration than the US.^40^ The decrease over time in the role of the NIH is congruent with the decrease in the proportion of R&D funding from government sources in the US over time. In the US, government sources accounted for 20% of R&D in 2021 (latest available data^41^), while they represented 32% in 2010^42^ and 57% in the 1970s.^43^ In 2025, there have been major threats for further sharp reductions in federal funding and increased uncertainty. Conversely, the proportion of R&D with government sources is higher in China and the overall R&D expenditure in China was very close to that of the US in 2021 and may have fully matched the US by now, if the long-term trends had continued in the last 4 years.^41^

### Limitations

Some limitations should be acknowledged. First, citations are not equivalent to disruptive innovation. However, it is difficult to measure disruption objectively, and even more so in recent work. There is some worrisome,^44^ but debated^45^ evidence for the substantial recent decline in disruptiveness. Second, the citation potential of recently published articles has not yet been fully realized. Moreover, given the substantially longer time available to be cited, articles published early in 2023 have major advantages to reach the top-cited list than those published in late 2024. However, the relative timing of publication within 2023-2024 is unlikely to be affected by country of provenance, type of study, or funding pattern. There is no evidence that articles from a specific country, type, or funding are more likely to start acquiring numerous citations late, while being relatively unnoticed early after publication.^46^ Such articles, called “sleeping beauties” to characterize their late citation blossoming, are rather uncommon.^46^ Finally, the extremely cited articles examined here represent less than 0.01% of the biomedical articles published. Given that other analyses have shown that China has recently overtaken the US in authorship of the top 1% most-cited articles,^5^ it is possible that the percentage of the US share would have diminished if a larger sample (eg, the top 1000 or 10 000 articles) had been chosen.

## Conclusions

Acknowledging these caveats, the analyzed publications offer key referential landmarks for large, prolific, and influential fields. This applies not only to traditional primary basic science research but also to centralized resources, new methods, high-impact randomized clinical trials, and even consensus statements, statistics articles, and reviews. In the current landscape, although the US remains a key player, its influence has diminished. The decrease is even more prominent for the presence of NIH funding and very prominent for solitary NIH funding. Strong public support of science is indispensable to ensure that research retains an orientation toward enhancing the public good. With threats of major cuts in government funding in 2025, it is important to avoid science becoming a political battlefield and to secure bipartisan support for biomedical research.

## Notes

Supplement 1. **eTable 1.** 100 Top-Cited Biomedical Papers Published in 2003-2004 **eTable 2.** 100 Top-Cited Biomedical Papers Published in 2013-2014 **eTable 3.** 100 Top-Cited Biomedical Papers Published in 2023-2024

Supplement 2.
Data Sharing Statement
