# Supplementary material for: Provenance and Funding of Extremely Cited Biomedical Articles Published Between 2003 and 2024
Source: JAMA Health Forum. 2025 Sep 12;6(9):e253045. doi: 10.1001/jamahealthforum.2025.3045 (PMC12432630; doi:10.1001/jamahealthforum.2025.3045)
Supplement: Supplement 1. — eTable 1. 100 Top-Cited Biomedical Papers Published in 2003-2004 eTable 2. 100 Top-Cited Biomedical Papers Published in 2013-2014 eTable 3. 100 Top-Cited Biomedical Papers Published in 2023-2024 [file jamahealthforum-e253045-s001.pdf]

Supplemental Online Content

Ioannidis JPA. Provenance and funding of extremely cited biomedical articles published between 2003 and 2024. *JAMA Health Forum*. 2025;6(9):e253045. doi:10.1001/jamahealthforum.2025.3045

eTable 1. 100 Top cited biomedical papers published in 2003-4

eTable 2. 100 Top cited biomedical papers published in 2013-4

eTable 3. 100 Top cited biomedical papers published in 2023-4

This supplemental material has been provided by the author to give readers additional information about their work.

© 2025 Ioannidis JPA. *JAMA Health Forum*.

eTable 1. 100 Top cited biomedical papers published in 2003-4

Title	Type of paper	Year	Source	Cited	Country	Classified funding
Measuring inconsistency in meta-analyses	Resource	2003	BMJ	48466	UK	UK
MUSCLE: Multiple sequence alignment with high accuracy and high throughput	Resource	2004	Nucleic Acids Research	35453	USA	European
UCSF Chimera - A visualization system for exploratory research and analysis	Resource	2004	Journal of Computational Chemistry	34953	USA	NIH
Cytoscape: A software Environment for integrated models of biomolecular interaction networks	Resource	2003	Genome Research	34551	USA	NIH
MicroRNAs: Genomics, Biogenesis, Mechanism, and Function	Resource	2004	Cell	31906	USA	NIH/non-profit
MrBayes 3: Bayesian phylogenetic inference under mixed models	Resource	2003	Bioinformatics	27150	Sweden	NSF/Sweden
Coot: Model-building tools for molecular graphics	Resource	2004	Acta Crystallographica Section D	25964	UK	UK/societies
Classification of surgical complications: A new proposal with evaluation in a cohort of 6336 patients and results of a survey	Resource	2004	Annals of Surgery	25433	Switzerland	None
EEGLAB: An open source toolbox for analysis of single-trial EEG dynamics including independent component analysis	Resource	2004	Journal of Neuroscience Methods	17538	USA	NIH/non-profit/institution
The seventh report of the joint national committee on prevention, detection, evaluation, and treatment of high blood pressure: The JNC 7 report	Consensus	2003	JAMA	17348	USA	NIH
International physical activity questionnaire: 12-Country reliability and validity	Resource	2003	Medicine and Science in Sports and Exercise	15013	Australia	None
Development and testing of a general Amber force field	Resource	2004	Journal of Computational Chemistry	14870	USA	NIH

© 2025 Ioannidis JPA. *JAMA Health Forum*.

Qualitative content analysis in nursing research: Concepts, procedures and measures to achieve trustworthiness	Resource	2004	Nurse Education Today	13264	Sweden	None
Global Prevalence of Diabetes: Estimates for the year 2000 and projections for 2030	Reference statistics	2004	Diabetes Care	12676	UK	None
Seventh report of the Joint National Committee on Prevention, Detection, Evaluation, and Treatment of High Blood Pressure	Consensus	2003	Hypertension	11236	USA	NIH
Mfold web server for nucleic acid folding and hybridization prediction	Resource	2003	Nucleic Acids Research	11185	USA	NIH
MEGA3: Integrated software for Molecular Evolutionary Genetics Analysis and sequence alignment.	Resource	2004	Briefings in bioinformatics	10724	USA	NIH/NSF/non-profit
Advances in functional and structural MR image analysis and implementation as FSL	Resource	2004	NeuroImage	10689	UK	UK/for-profit/non-profit
Activating Mutations in the Epidermal Growth Factor Receptor Underlying Responsiveness of Non-Small-Cell Lung Cancer to Gefitinib	Article	2004	NEJM	10509	USA	NIH
Prejudice, Social Stress, and Mental Health in Lesbian, Gay, and Bisexual Populations: Conceptual Issues and Research Evidence	Article	2003	Psychological Bulletin	10327	USA	NIH
Bioconductor: open software development for computational biology and bioinformatics.	Resource	2004	Genome biology	9996	USA	None
Chronic kidney disease and the risks of death, cardiovascular events, and hospitalization	Article	2004	NEJM	9949	USA	NIH
The functions of animal microRNAs	Review	2004	Nature	9717	USA	NIH
Bevacizumab plus irinotecan, fluorouracil, and leucovorin for metastatic colorectal cancer	Article	2004	NEJM	9669	USA	NIH
Linear models and empirical bayes methods for assessing differential expression in microarray experiments	Resource	2004	Statistical Applications in Genetics and Molecular Biology	9650	Australia	Australia

© 2025 Ioannidis JPA. *JAMA Health Forum*.

WebLogo: A sequence logo generator	Resource	2004	Genome Research	9649	USA	NIH
Appropriate body-mass index for Asian populations and its implications for policy and intervention strategies	Article	2004	Lancet	9541	Switzerland	Japan/Singapore
Effect of potentially modifiable risk factors associated with myocardial infarction in 52 countries (the INTERHEART study): Case-control study	Article	2004	Lancet	9454	Canada	Canada/for-profit/non-profit
APE: Analyses of phylogenetics and evolution in R language	Resource	2004	Bioinformatics	9296	France	Germany
Prospective identification of tumorigenic breast cancer cells	Article	2003	PNAS	8961	USA	NIH
EGFR mutations in lung, cancer: Correlation with clinical response to gefitinib therapy	Article	2004	Science	8893	USA	NIH
Exploration, normalization, and summaries of high density oligonucleotide array probe level data.	Resource	2003	Biostatistics	8770	USA	NIH
The Benefits of Being Present: Mindfulness and Its Role in Psychological Well-Being	Review	2003	Journal of Personality and Social Psychology	8530	USA	NIH/societies/non-profit/Canada
Essential oils: Their antibacterial properties and potential applications in foods - A review	Review	2004	International Journal of Food Microbiology	8444	Netherlands	None
The biology of VEGF and its receptors	Review	2003	Nature Medicine	8424	USA	None
Staging of brain pathology related to sporadic Parkinson's disease	Article	2003	Neurobiology of Aging	8338	Germany	Germany/Australia
Obesity is associated with macrophage accumulation in adipose tissue	Article	2003	Journal of Clinical Investigation	8216	USA	NIH
The International Classification of Headache Disorders: 2nd edition.	Consensus	2004	Cephalalgia	8168	Denmark	None
DAVID: Database for Annotation, Visualization, and Integrated Discovery.	Resource	2003	Genome biology	8052	USA	NIH

© 2025 Ioannidis JPA. *JAMA Health Forum*.

Individual Differences in Two Emotion Regulation Processes: Implications for Affect, Relationships, and Well-Being	Article	2003	Journal of Personality and Social Psychology	7851	USA	NIH
Glide: A New Approach for Rapid, Accurate Docking and Scoring. 1. Method and Assessment of Docking Accuracy	Resource	2004	Journal of Medicinal Chemistry	7775	USA	NIH
Neutrophil Extracellular Traps Kill Bacteria	Article	2004	Science	7735	Germany	NIH
Statistical significance for genomewide studies	Article	2003	PNAS	7684	USA	None
PGC-1α-responsive genes involved in oxidative phosphorylation are coordinately downregulated in human diabetes	Article	2003	Nature Genetics	7474	USA	HHMI/non-profit/for-profit/Sweden
A Modified Poisson Regression Approach to Prospective Studies with Binary Data	Resource	2004	American Journal of Epidemiology	7234	Canada	Canada
Control of regulatory T cell development by the transcription factor Foxp3	Article	2003	Science	7173	Japan	Japan
Toll-like receptor signalling	Review	2004	Nature Reviews Immunology	7155	Japan	Japan/non-profit
Development of a new Resilience scale: The Connor-Davidson Resilience scale (CD-RISC)	Resource	2003	Depression and Anxiety	7048	USA	NIH/for-profit
MUSCLE: A multiple sequence alignment method with reduced time and space complexity	Resource	2004	BMC Bioinformatics	6960	USA	Japan
The Epidemiology of Major Depressive Disorder: Results from the National Comorbidity Survey Replication (NCS-R)	Article	2003	JAMA	6817	USA	NIH
Inference of population structure using multilocus genotype data: Linked loci and correlated allele frequencies	Article	2003	Genetics	6812	Germany	NIH
A comparison of normalization methods for high density oligonucleotide array data based on variance and bias	Resource	2003	Bioinformatics	6728	USA	None
Influence of life stress on depression: Moderation by a polymorphism in the 5-HTT gene	Article	2003	Science	6536	UK	NIH

© 2025 Ioannidis JPA. *JAMA Health Forum*.

Foxp3 programs the development and function of CD4+CD25+ regulatory T cells	Article	2003	Nature Immunology	6509	USA	NIH/HHMI/non-profit
Polyphenols: Food sources and bioavailability	Review	2004	American Journal of Clinical Nutrition	6503	France	None
Overweight, obesity, and mortality from cancer in a prospectively studied cohort of U.S. Adults	Article	2003	NEJM	6472	USA	None
Identification of human brain tumour initiating cells	Article	2004	Nature	6462	Canada	Canada/non-profit/institution
Mild cognitive impairment as a diagnostic entity	Review	2004	Journal of Internal Medicine	6296	USA	NIH
Network biology: Understanding the cell's functional organization	Review	2004	Nature Reviews Genetics	6269	USA	NIH/NSF/DOE
The fourth report on the diagnosis, evaluation, and treatment of high blood pressure in children and adolescents	Consensus	2004	Pediatrics	6202	USA	NIH
Multidimensional Assessment of Emotion Regulation and Dysregulation: Development, Factor Structure, and Initial Validation of the Difficulties in Emotion Regulation Scale	Resource	2004	Journal of Psychopathology and Behavioral Assessment	6099	USA	Institution
MicroRNAs: Small RNAs with a big role in gene regulation	Review	2004	Nature Reviews Genetics	6068	USA	NIH
The mirror-neuron system	Review	2004	Annual Review of Neuroscience	6045	Italy	None
Confidence limits for the indirect effect: Distribution of the product and resampling methods	Resource	2004	Multivariate Behavioral Research	6012	USA	NIH
Normalization of real-time quantitative reverse transcription-PCR data: A model-based variance estimation approach to identify genes suited for normalization, applied to bladder and colon cancer data sets	Article	2004	Cancer Research	5955	Denmark	European
Mass spectrometry-based proteomics	Review	2003	Nature	5925	USA	NIH/for-profit/Denmark

© 2025 Ioannidis JPA. *JAMA Health Forum*.

Photodynamic therapy for cancer	Review	2003	Nature Reviews Cancer	5914	USA	NIH/non-profit
Bariatric surgery: A systematic review and meta-analysis	Review	2004	JAMA	5849	USA	For-profit
Targeting HIF-1 for cancer therapy	Review	2003	Nature Reviews Cancer	5789	USA	None
Improved prediction of signal peptides: SignalP 3.0	Resource	2004	Journal of Molecular Biology	5767	Denmark	Denmark/for-profit
Acute renal failure - definition, outcome measures, animal models, fluid therapy and information technology needs: the Second International Consensus Conference of the Acute Dialysis Quality Initiative (ADQI) Group.	Consensus	2004	Critical care (London, England)	5694	Australia	None
Chronic inflammation in fat plays a crucial role in the development of obesity-related insulin resistance	Article	2003	Journal of Clinical Investigation	5600	USA	None
Markers of inflammation and cardiovascular disease: Application to clinical and public health practice: A statement for healthcare professionals from the centers for disease control and prevention and the American Heart Association	Consensus	2003	Circulation	5586	USA	CDC/For-profit
Bacterial biofilms: From the natural environment to infectious diseases	Review	2004	Nature Reviews Microbiology	5585	USA	NIH
Methodological index for non-randomized studies (Minors): Development and validation of a new instrument	Resource	2003	ANZ Journal of Surgery	5548	France	None
Osteoclast differentiation and activation	Review	2003	Nature	5522	USA	None
DnaSP, DNA polymorphism analyses by the coalescent and other methods	Resource	2003	Bioinformatics	5457	Spain	Spain
ARB: A software environment for sequence data	Resource	2004	Nucleic Acids Research	5455	Germany	Germany

© 2025 Ioannidis JPA. *JAMA Health Forum*.

Revised 2003 consensus on diagnostic criteria and long-term health risks related to polycystic ovary syndrome	Consensus	2004	Fertility and Sterility	5445	Netherland	For-profit/societies
Revised 2003 consensus on diagnostic criteria and long-term health risks related to polycystic ovary syndrome	Consensus	2004	Human Reproduction	5398	Netherlands	For-profit/societies
Silver nanoparticles as antimicrobial agent: A case study on E. coli as a model for Gram-negative bacteria	Article	2004	Journal of Colloid and Interface Science	5352	Croatia	Croatia
Functional connectivity in the resting brain: A network analysis of the default mode hypothesis	Article	2003	PNAS	5325	USA	NIH
Epidemiologic classification of human papillomavirus types associated with cervical cancer	Article	2003	NEJM	5297	Spain	Spain
Docetaxel plus prednisone or mitoxantrone plus prednisone for advanced prostate cancer	Article	2004	NEJM	5269	Canada	For-profit
A multigene assay to predict recurrence of tamoxifen-treated, node-negative breast cancer	Article	2004	NeEJM	5252	USA	NIH/for-profit
Preoperative versus postoperative chemoradiotherapy for rectal cancer	Article	2004	NEJM	5249	Germany	Germany
Mindfulness-based interventions in context: Past, present, and future	Review	2003	Clinical Psychology: Science and Practice	5210	USA	None
Implications of recent clinical trials for the National Cholesterol Education Program Adult Treatment Panel III guidelines	Consensus	2004	Circulation	5198	USA	NIH/societies
The international HapMap project	Resource	2003	Nature	5184	UK	NIH/non-profit/for-profit/international/institutional
The chemokine system in diverse forms of macrophage activation and polarization	Review	2004	Trends in Immunology	5175	Italy	Italy
Mechanisms of TGF-β signaling from cell membrane to the nucleus	Review	2003	Cell	5153	USA	NIH/HHMI

© 2025 Ioannidis JPA. *JAMA Health Forum*.

Brown Adipose Tissue: Function and Physiological Significance	Review	2004	Physiological Reviews	5152	Sweden	Sweden
The epidemiology of sepsis in the United States from 1979 through 2000	Article	2003	NEJM	5131	USA	NIH
Alternative activation of macrophages	Review	2003	Nature Reviews Immunology	5121	UK	UK/non-profit
Cardiac-Resynchronization Therapy with or without an Implantable Defibrillator in Advanced Chronic Heart Failure	Article	2004	NEJM	5104	USA	For-profit
Epigenetic regulation of gene expression: How the genome integrates intrinsic and environmental signals	Review	2003	Nature Genetics	5062	USA	None
The gut microbiota as an environmental factor that regulates fat storage	Article	2004	PNAS	5048	USA	NIH
Hazards of heavy metal contamination	Review	2003	British Medical Bulletin	5037	UK	None
2001 SCCM/ESICM/ACCP/ATS/SIS International Sepsis Definitions Conference	Consensus	2003	Critical Care Medicine	5008	USA	Societies
SIFT: Predicting amino acid changes that affect protein function	Resource	2003	Nucleic Acids Research	4983	USA	NIH

© 2025 Ioannidis JPA. *JAMA Health Forum*.

eTable 2. 100 Top-cited biomedical papers published in 2013-4

Title	Type of paper	Year	Source title	Cited	Country	Funding
Moderated estimation of fold change and dispersion for RNA-seq data with DESeq2	Resource	2014	Genome Biology	53866	Germany	NIH/European/institution
Trimmomatic: A flexible trimmer for Illumina sequence data	Resource	2014	Bioinformatics	41694	Germany	Germany
MEGA6: Molecular evolutionary genetics analysis version 6.0	Resource	2013	Molecular Biology and Evolution	36548	USA	NIH/Japan
MAFFT multiple sequence alignment software version 7: Improvements in performance and usability	Resource	2013	Molecular Biology and Evolution	30829	Japan	Japan
STAR: Ultrafast universal RNA-seq aligner	Resource	2013	Bioinformatics	29530	USA	NIH
RAxML version 8: A tool for phylogenetic analysis and post-analysis of large phylogenies	Resource	2014	Bioinformatics	24870	Germany	Institution
The SILVA ribosomal RNA gene database project: Improved data processing and web-based tools	Resource	2013	Nucleic Acids Research	21199	Germany	Germany
FeatureCounts: An efficient general purpose program for assigning sequence reads to genomic features	Resource	2014	Bioinformatics	14943	USA	Australia
Investigation of the freely available easy-to-use software 'EZR' for medical statistics	Resource	2013	Bone Marrow Transplantation	13173	Japan	None listed
UPARSE: Highly accurate OTU sequences from microbial amplicon reads	Resource	2013	Nature Methods	12968	USA	Non-profit/institution
Phyloseq: An R Package for Reproducible Interactive Analysis and Graphics of Microbiome Census Data	Resource	2013	PLoS ONE	12749	USA	NIH
Multiplex genome engineering using CRISPR/Cas systems	Article	2013	Science	12157	USA	NIH
Cancer statistics, 2013	Reference statistics	2013	CA Cancer	11912	USA	Societies
Cancer statistics, 2014	Reference statistics	2014	CA Cancer	11514	USA	Societies
Prokka: Rapid prokaryotic genome annotation	Resource	2014	Bioinformatics	11508	Australia	Institution

© 2025 Ioannidis JPA. *JAMA Health Forum*.

Integrative analysis of complex cancer genomics and clinical profiles using the cBioPortal	Resource	2013	Science Signaling	11145	USA	NIH
The hallmarks of aging	Review	2013	Cell	10740	Spain	Multiple international/non-profit/for-profit
TopHat2: Accurate alignment of transcriptomes in the presence of insertions, deletions and gene fusions	Resource	2013	Genome Biology	9853	USA	NIH
Global, regional, and national prevalence of overweight and obesity in children and adults during 1980-2013: A systematic analysis for the Global Burden of Disease Study 2013	Reference statistics	2014	Lancet	9318	USA	NIH/for-profit/non-profit/international
Executive functions	Review	2013	Annual Review of Psychology	8721	Canada	NIH
GSVA: Gene set variation analysis for microarray and RNA-Seq data	Resource	2013	BMC Bioinformatics	8442	Spain	NIH/Spain
Genome engineering using the CRISPR-Cas9 system	Article	2013	Nature Protocols	8184	USA	NIH/non-profit
RNA-guided human genome engineering via Cas9	Article	2013	Science	7500	USA	NIH
Signatures of mutational processes in human cancer	Article	2013	Nature	7461	UK	NIH/European
Random effects structure for confirmatory hypothesis testing: Keep it maximal	Article	2013	Journal of Memory and Language	7436	UK	NIH/UK
Diet rapidly and reproducibly alters the human gut microbiome	Article	2014	Nature	7344	USA	NIH
NCBI GEO: Archive for functional genomics data sets – Update	Resource	2013	Nucleic Acids Research	7202	USA	NIH
Predictive functional profiling of microbial communities using 16S rRNA marker gene sequences	Article	2013	Nature Biotechnology	7186	USA	NIH/NSF/ARO/non-profit/Canada
The global distribution and burden of dengue	Reference statistics	2013	Nature	7062	UK	NIH/DHS/non-profit/European
Estimating the sample mean and standard deviation from the sample size, median, range and/or interquartile range	Resource	2014	BMC Medical Research Methodology	6971	Hong Kong	Hong Kong/institution
The strengthening the reporting of observational studies in epidemiology (STROBE) statement: Guidelines for reporting observational studies	Consensus	2014	International Journal of Surgery	6842	Switzerland	European

© 2025 Ioannidis JPA. *JAMA Health Forum*.

Prevalence of childhood and adult obesity in the United States, 2011-2012	Reference statistics	2014	JAMA	6701	USA	CDC
Expert consensus document: The international scientific association for probiotics and prebiotics consensus statement on the scope and appropriate use of the term probiotic	Consensus	2014	Nature Reviews Gastroenterology and Hepatology	6695	USA	Societies
2014 Evidence-based guideline for the management of high blood pressure in adults: Report from the panel members appointed to the Eighth Joint National Committee (JNC 8)	Consensus	2014	JAMA	6601	USA	NIH
Frailty in elderly people	Review	2013	Lancet	6553	UK	None listed
Molecular mechanisms of epithelial-mesenchymal transition	Review	2014	Nature Reviews Molecular Cell Biology	6501	USA	NIH
Natural RNA circles function as efficient microRNA sponges	Article	2013	Nature	6496	Denmark	Denmark/non-profit
Standards for reporting qualitative research: A synthesis of recommendations	Consensus	2014	Academic Medicine	6490	USA	None listed
Using the framework method for the analysis of qualitative data in multi-disciplinary health research	Resource	2013	BMC Medical Research Methodology	6488	UK	UK
Circular RNAs are a large class of animal RNAs with regulatory potency	Article	2013	Nature	6481	Germany	Germany/institution
QUAST: Quality assessment tool for genome assemblies	Resource	2013	Bioinformatics	6456	Russia	NIH
Extracellular vesicles: Exosomes, microvesicles, and friends	Review	2013	Journal of Cell Biology	6455	France	France/for-profit/institution
Better reporting of interventions: Template for intervention description and replication (TIDieR) checklist and guide	Consensus	2014	BMJ	6214	Australia	UK/Australia/non-profit
The International Classification of Headache Disorders, 3rd edition (beta version)	Consensus	2013	Cephalalgia	6190	USA	NIH/societies/for-profit/institution/Canada

© 2025 Ioannidis JPA. *JAMA Health Forum*.

Integrative Genomics Viewer (IGV): High-performance genomics data visualization and exploration	Resource	2013	Briefings in Bioinformatics	6170	USA	NIH
Evaluation of general 16S ribosomal RNA gene PCR primers for classical and next-generation sequencing-based diversity studies	Article	2013	Nucleic Acids Research	6068	Germany	Germany/Austria/European
Inferring tumour purity and stromal and immune cell admixture from expression data	Article	2013	Nature Communications	6055	USA	NIH
GROMACS 4.5: A high-throughput and highly parallel open source molecular simulation toolkit	Resource	2013	Bioinformatics	6052	Sweden	NIH/European/Sweden
De novo transcript sequence reconstruction from RNA-seq using the Trinity platform for reference generation and analysis	Resource	2013	Nature Protocols	6018	USA	NIH/NSF/non-profit/for-profit/international
Cancer genome landscapes	Review	2013	Science	6010	USA	NIH
Pilon: An integrated tool for comprehensive microbial variant detection and genome assembly improvement	Resource	2014	PLoS ONE	6001	USA	NIH
Microenvironmental regulation of tumor progression and metastasis	Review	2013	Nature Medicine	5957	USA	NIH/societies/non-profit/Canada
Biological insights from 108 schizophrenia-associated genetic loci	Article	2014	Nature	5906	UK	NIH/VA/UK/European
The cancer genome atlas pan-cancer analysis project	Resource	2013	Nature Genetics	5771	USA	NIH
InterProScan 5: Genome-scale protein function classification	Resource	2014	Bioinformatics	5554	UK	European
Projecting cancer incidence and deaths to 2030: The unexpected burden of thyroid, liver, and pancreas cancers in the united states	Reference statistics	2014	Cancer Research	5482	USA	Non-profit
3D bioprinting of tissues and organs	Review	2014	Nature Biotechnology	5441	USA	None listed
Content analysis and thematic analysis: Implications for conducting a qualitative descriptive study	Review	2013	Nursing and Health Sciences	5418	Finland	Institution
PD-1 blockade induces responses by inhibiting adaptive immune resistance	Article	2014	Nature	5384	USA	NIH/HHMI/for-profit/non-profit/societies
2013 ACCF/AHA guideline for the management of heart failure: A report of the American college of	Consensus	2013	Journal of the American	5380	USA	Societies

© 2025 Ioannidis JPA. *JAMA Health Forum*.

cardiology foundation/american heart association task force on practice guidelines			College of Cardiology			
Maternal and child undernutrition and overweight in low-income and middle-income countries	Review	2013	Lancet	5374	USA	Non-profit
A 3D map of the human genome at kilobase resolution reveals principles of chromatin looping	Article	2014	Cell	5346	USA	NIH/NSF/non-profit/institution
Angiotensin-neprilysin inhibition versus enalapril in heart failure	Article	2014	NEJM	5325	USA/UK	For-profit
Increased survival in pancreatic cancer with nab-paclitaxel plus gemcitabine	Article	2013	NEJM	5254	USA	NIH
Genome sequence-based species delimitation with confidence intervals and improved distance functions	Article	2013	BMC Bioinformatics	5249	Germany	Germany/Spain
Development of a dual-index sequencing strategy and curation pipeline for analyzing amplicon sequence data on the miseq illumina sequencing platform	Resource	2013	Applied and Environmental Microbiology	5161	USA	NIH
2012 Revised International Chapel Hill consensus conference nomenclature of vasculitides	Consensus	2013	Arthritis and Rheumatism	5161	USA	None listed
Regulation of ferroptotic cancer cell death by GPX4	Article	2014	Cell	5127	USA	NIH
Power failure: Why small sample size undermines the reliability of neuroscience	Article	2013	Nature Reviews Neuroscience	5098	UK	UK
Global prevalence of glaucoma and projections of glaucoma burden through 2040: A systematic review and meta-analysis	Reference statistics	2014	Ophthalmology	5070	Singapore	Singapore
Oncology meets immunology: The cancer-immunity cycle	Review	2013	Immunity	5060	USA	None listed
ROS function in redox signaling and oxidative stress	Review	2014	Current Biology	5059	USA	NIH
BEAST 2: A Software Platform for Bayesian Evolutionary Analysis	Resource	2014	PLoS Computational Biology	5032	New Zealand	NIH
Classification of acute pancreatitis - 2012: Revision of the Atlanta classification and definitions by international consensus	Consensus	2013	Gut	4963	USA	Societies
Comprehensive molecular characterization of gastric adenocarcinoma	Article	2014	Nature	4953	USA	NIH

© 2025 Ioannidis JPA. *JAMA Health Forum*.

Deciphering key features in protein structures with the new ENDscript server	Resource	2014	Nucleic Acids Research	4933	France	France
The carbohydrate-active enzymes database (CAZy) in 2013	Resource	2014	Nucleic Acids Research	4892	France	France
The behavior change technique taxonomy (v1) of 93 hierarchically clustered techniques: Building an international consensus for the reporting of behavior change interventions	Consensus	2013	Annals of Behavioral Medicine	4862	UK	UK
Biogenesis, secretion, and intercellular interactions of exosomes and other extracellular vesicles	Review	2014	Annual Review of Cell and Developmental Biology	4848	France	France/non-profit
Pfam: The protein families database	Review	2014	Nucleic Acids Research	4846	USA	HHMI/European/non-profit
Surviving sepsis campaign: International guidelines for management of severe sepsis and septic shock: 2012	Consensus	2013	Critical Care Medicine	4840	USA	Non-profit
Heart Disease and Stroke Statistics - 2014 Update: A report from the American Heart Association	Review	2014	Circulation	4810	USA	NIH
The new frontier of genome engineering with CRISPR-Cas9	Review	2014	Science	4775	USA/Germany	NIH/NSF/non-profit
Analysis of the structural diversity, substitution patterns, and frequency of nitrogen heterocycles among U.S. FDA approved pharmaceuticals	Article	2014	Journal of Medicinal Chemistry	4603	USA	NSF
Enrichr: Interactive and collaborative HTML5 gene list enrichment analysis tool	Resource	2013	BMC Bioinformatics	4601	USA	NIH
From fastQ data to high-confidence variant calls: The genome analysis toolkit best practices pipeline	Consensus	2013	Current Protocols in Bioinformatics	4600	USA	NIH
Macrophage Activation and Polarization: Nomenclature and Experimental Guidelines	Consensus	2014	Immunity	4574	USA	NIH/UK/European/non-profit
Heart disease and stroke statistics-2013 update: A Report from the American Heart Association	Reference statistics	2013	Circulation	4572	USA	NIH
Diagnosis and classification of diabetes mellitus	Consensus	2014	Diabetes Care	4568	USA	Societies

© 2025 Ioannidis JPA. *JAMA Health Forum*.

Toxicity, mechanism and health effects of some heavy metals	Review	2014	Interdisciplinary Toxicology	4563	India	None listed
SPIRIT 2013 statement: Defining standard protocol items for clinical trials	Consensus	2013	Annals of Internal Medicine	4545	Canada	Canada
Global burden of disease attributable to mental and substance use disorders: Findings from the Global Burden of Disease Study 2010	Reference statistics	2013	Lancet	4534	Australia	NIH/Australia
Ultrasensitive fluorescent proteins for imaging neuronal activity	Article	2013	Nature	4512	USA	NIH/Switzerland/European
A general framework for estimating the relative pathogenicity of human genetic variants	Article	2014	Nature Genetics	4510	USA	NIH
Regulation of microRNA biogenesis	Review	2014	Nature Reviews Molecular Cell Biology	4458	Korea	Korea
Ultrasensitive photodetectors based on monolayer MoS 2	Article	2013	Nature Nanotechnology	4446	Switzerland	Switzerland/European
Chimeric antigen receptor T cells for sustained remissions in leukemia	Article	2014	NEJM	4414	USA	NIH/for-profit/non-profit/institution
Development and applications of CRISPR-Cas9 for genome engineering	Review	2014	Cell	4403	USA	NIH/NSF/non-profit
Cancer incidence and mortality patterns in Europe: Estimates for 40 countries in 2012	Reference statistics	2013	European Journal of Cancer	4381	France	European/IARC
Lipid peroxidation: Production, metabolism, and signaling mechanisms of malondialdehyde and 4-hydroxy-2-nonenal	Review	2014	Oxidative Medicine and Cellular Longevity	4376	Spain	Spain

© 2025 Ioannidis JPA. *JAMA Health Forum*.

eTable 3. 100 Top cited biomedical papers published in 2023-4

Title	Type of paper	Year	Source title	Cited	Country	Funding
Cancer statistics, 2023	Reference statistics	2023	CA Cancer	9785	USA	Societies
Global cancer statistics 2022: GLOBOCAN estimates of incidence and mortality worldwide for 36 cancers in 185 countries	Reference statistics	2024	CA Cancer	3306	France	IARC+WHO
Cancer statistics, 2024	Reference statistics	2024	CA Cancer	2543	USA	Societies
UniProt: the Universal Protein Knowledgebase in 2023	Resources	2023	Nucleic Acids Research	2494	UK	NIH/UK/Germany/European
KEGG for taxonomy-based analysis of pathways and genomes	Resources	2023	Nucleic Acids Research	2282	Japan	None
Heart Disease and Stroke Statistics - 2023 Update: A Report from the American Heart Association	Reference statistics	2023	Circulation	2199	USA	NIH/societies
The STRING database in 2023: protein-protein association networks and functional enrichment analyses for any sequenced genome of interest	Resources	2023	Nucleic Acids Research	2130	Switzerland/Denmark/ Germany	Multiple international/for-profit
Lecanemab in Early Alzheimer's Disease	Article	2023	NEJM	2101	USA	For-profit
Long COVID: major findings, mechanisms and recommendations	Review	2023	Nature Reviews Microbiology	1889	USA	NIH
Hallmarks of aging: An expanding universe	Review	2023	Cell	1810	Spain/France	For-profit/non-profit/international/institution
Performance of ChatGPT on USMLE: Potential for AI-assisted medical education using large language models	Article	2023	PLOS Digital Health	1607	USA	None

© 2025 Ioannidis JPA. *JAMA Health Forum*.

The SCARE 2023 guideline: updating consensus Surgical CAse REport (SCARE) guidelines	Consensus	2023	International journal of surgery	1593	UK	None
FinnGen provides genetic insights from a well-phenotyped isolated population	Article	2023	Nature	1424	Finland	Finland
2023 Alzheimer's disease facts and figures	Review	2023	Alzheimer's and Dementia	1372	USA	CDC/non-profit/institution
Swin-Unet: Unet-Like Pure Transformer for Medical Image Segmentation	Resources	2023	Lecture Notes in Artificial Intelligence and Lecture Notes in Bioinformatics)	1364	China	None
2023 ESC Guidelines for the management of acute coronary syndromes	Consensus	2023	European Heart Journal	1348	Ireland/Spain	Societies
PubChem 2023 update	Resources	2023	Nucleic Acids Research	1298	USA	NIH
Colorectal cancer statistics, 2023	Reference statistics	2023	CA Cancer	1213	USA	Societies
2023 ESH Guidelines for the management of arterial hypertension the Task Force for the management of arterial hypertension of the European Society of Hypertension: Endorsed by the International Society of Hypertension (ISH) and the European Renal Association (ERA)	Consensus	2023	Journal of Hypertension	1207	Italy/Germany	Societies
Global, regional, and national burden of diabetes from 1990 to 2021, with projections of prevalence	Reference statistics	2023	Lancet	1206	USA	NIH/non-profit/multiple international

© 2025 Ioannidis JPA. *JAMA Health Forum*.

to 2050: a systematic analysis for the Global Burden of Disease Study 2021						
ChatGPT Utility in Healthcare Education, Research, and Practice: Systematic Review on the Promising Perspectives and Valid Concerns	Review	2023	Healthcare (Switzerland)	1189	Jordan	None
Covid-19 pandemic and online learning: the challenges and opportunities	Review	2023	Interactive Learning Environments	1181	Cyprus	None
Accurate structure prediction of biomolecular interactions with AlphaFold 3	Resources	2024	Nature	1146	UK	For-profit
A multisociety Delphi consensus statement on new fatty liver disease nomenclature	Consensus	2023	Journal of Hepatology	1117	USA	European/societies
2. Classification and Diagnosis of Diabetes: Standards of Care in Diabetes—2023	Consensus	2023	Diabetes Care	1113	USA	Societies
InterPro in 2022	Resources	2023	Nucleic Acids Research	1112	UK	NIH/NSF/non-profit/multiple international
Empagliflozin in Patients with Chronic Kidney Disease	Article	2023	New England Journal of Medicine	1104	UK	Non-profit/for-profit/UK/Japan
Evolutionary-scale prediction of atomic-level protein structure with a language model	Article	2023	Science	1095	USA	Non-profit/for-profit/UK/Japan
The evolving tumor microenvironment: From cancer initiation to metastatic outgrowth	Review	2023	Cancer Cell	997	Netherlands/Switzerland	For-profit/non-profit/international/institution
A multisociety Delphi consensus statement on new fatty liver disease nomenclature	Consensus	2023	Hepatology	957	USA/UK	None

© 2025 Ioannidis JPA. *JAMA Health Forum*.

Prevalence and Characteristics of Autism Spectrum Disorder Among Children Aged 8 Years — Autism and Developmental Disabilities Monitoring Network, 11 Sites, United States, 2020	Article	2023	MMWR Surveillance Summaries	953	USA	None
The global epidemiology of nonalcoholic fatty liver disease (NAFLD) and nonalcoholic steatohepatitis (NASH): a systematic review	Review	2023	Hepatology	947	USA	For-profit
How Does ChatGPT Perform on the United States Medical Licensing Examination? The Implications of Large Language Models for Medical Education and Knowledge Assessment	Article	2023	JMIR Medical Education	929	USA	NIH/institution
Large language models in medicine	Review	2023	Nature Medicine	887	Singapore	Singapore/international
AASLD Practice Guidance on the clinical assessment and management of nonalcoholic fatty liver disease	Consensus	2023	Hepatology	857	USA	Societies
Comparing Physician and Artificial Intelligence Chatbot Responses to Patient Questions Posted to a Public Social Media Forum	Article	2023	JAMA Internal Medicine	852	USA	NIH/non-profit
2022 ESC/ERS Guidelines for the diagnosis and treatment of pulmonary hypertension	Consensus	2023	European Respiratory Journal	793	Germany/Belgium	Societies
TBtools-II: A “one for all, all for one” bioinformatics platform for biological big-data mining	Resources	2023	Molecular Plant	789	China	China
Large language models encode clinical knowledge	Article	2023	Nature	784	USA	For-profit

© 2025 Ioannidis JPA. *JAMA Health Forum*.

Donanemab in Early Symptomatic Alzheimer Disease: The TRAILBLAZER-ALZ 2 Randomized Clinical Trial	Article	2023	JAMA	757	USA	NIH/for-profit/societies
Long non-coding RNAs: definitions, functions, challenges and recommendations	Review	2023	Nature Reviews Molecular Cell Biology	747	Australia	Japan
The IPD-IMGT/HLA Database	Resources	2023	Nucleic Acids Research	737	UK	Societies/for-profit/non-profit/institution
Benefits, Limits, and Risks of GPT-4 as an AI Chatbot for Medicine.	Review	2023	NEJM	732	USA	For-profit
Semaglutide and Cardiovascular Outcomes in Obesity without Diabetes	Article	2023	NEJM	719	USA	For-profit
AntiSMASH 7.0: New and improved predictions for detection, regulation, chemical structures and visualisation	Resources	2023	Nucleic Acids Research	719	Denmark/Netherlands	For-profit/multiple international
The Gene Ontology knowledgebase in 2023	Resources	2023	Genetics	719	USA	NIH/NSF/DOE/non-profit/multiple European
2023 Focused Update of the 2021 ESC Guidelines for the diagnosis and treatment of acute and chronic heart failure Developed by the task force for the diagnosis and treatment of acute and chronic heart failure of the European Society of Cardiology (ESC) With the special contribution of the Heart Failure Association (HFA) of the ESC	Consensus	2023	European Heart Journal	715	UK/Italy	Societies
2023 ESC Guidelines for the management of cardiomyopathies: Developed by the task force on the	Consensus	2023	European Heart Journal	713	Spain/UK	Societies

© 2025 Ioannidis JPA. *JAMA Health Forum*.

management of cardiomyopathies of the European Society of Cardiology (ESC)						
Minimal information for studies of extracellular vesicles (MISEV2023): From basic to advanced approaches	Consensus	2024	Journal of Extracellular Vesicles	712	USA/France/Ireland/UK	None
Global burden of colorectal cancer in 2020 and 2040: Incidence and mortality estimates from GLOBOCAN	Reference statistics	2023	Gut	708	France	IARC
Metastatic colorectal cancer: ESMO Clinical Practice Guideline for diagnosis, treatment and follow-up ☆	Consensus	2023	Annals of Oncology	671	Switzerland	Societies
SARS-CoV-2 variant biology: immune escape, transmission and fitness	Review	2023	Nature Reviews Microbiology	670	UK	UK/non-profit
2023 ACC/AHA/ACCP/HRS Guideline for the Diagnosis and Management of Atrial Fibrillation: A Report of the American College of Cardiology/American Heart Association Joint Committee on Clinical Practice Guidelines	Consensus	2024	Circulation	628	USA	Societies
Clinical Practice Guideline for the Evaluation and Treatment of Children and Adolescents with Obesity	Consensus	2023	Pediatrics	588	USA	Societies
KDIGO 2024 Clinical Practice Guideline for the Evaluation and Management of Chronic Kidney Disease	Consensus	2024	Kidney International	578	Unclear	Societies
UCSF ChimeraX: Tools for structure building and analysis	Resources	2023	Protein Science	576	USA	NIH/non-profit

© 2025 Ioannidis JPA. *JAMA Health Forum*.

Global burden of liver disease: 2023 update	Reference statistics	2023	Journal of Hepatology	575	USA	Institutional/Chile
YaHS: yet another Hi-C scaffolding tool	Resources	2023	Bioinformatics	570	UK	Non-profit
Global estimates of incidence and mortality of cervical cancer in 2020: a baseline analysis of the WHO Global Cervical Cancer Elimination Initiative	Reference statistics	2023	Lancet Global Health	556	France	European
9. Pharmacologic Approaches to Glycemic Treatment: Standards of Care in Diabetes—2023	Consensus	2023	Diabetes Care	556	USA	Societies
CARD 2023: expanded curation, support for machine learning, and resistome prediction at the Comprehensive Antibiotic Resistance Database	Resources	2023	Nucleic Acids Research	533	Canada	Canada/institution/for-profit
Reactive oxygen species, toxicity, oxidative stress, and antioxidants: chronic diseases and aging	Review	2023	Archives of Toxicology	533	Slovakia	Slovakia
The NHGRI-EBI GWAS Catalog: knowledgebase and deposition resource	Resources	2023	Nucleic Acids Research	529	UK	NIH/non-profit/multiple international
Alarming antibody evasion properties of rising SARS-CoV-2 BQ and XBB subvariants	Article	2023	Cell	523	USA	NIH/non-profit/for-profit/institution
Hallmarks of neurodegenerative diseases	Review	2023	Cell	520	Belgium	NIH/non-profit/multiple international
Revolutionizing healthcare: the role of artificial intelligence in clinical practice	Review	2023	BMC Medical Education	512	Saudi Arabia	None
The impact of COVID-19 lockdown on child and adolescent mental health: systematic review	Review	2023	European Child and Adolescent Psychiatry	509	UK	Spain/non-profit/institution

© 2025 Ioannidis JPA. *JAMA Health Forum*.

Evaluating the Feasibility of ChatGPT in Healthcare: An Analysis of Multiple Clinical and Research Scenarios	Article	2023	Journal of Medical Systems	504	Italy	Institution
Lobar or Sublobar Resection for Peripheral Stage IA Non-Small-Cell Lung Cancer	Article	2023	New England Journal of Medicine	494	USA	NIH/for-profit
2023 ESC Guidelines for the management of endocarditis	Consensus	2023	European Heart Journal	493	Spain/Germany	Societies
Foundation models for generalist medical artificial intelligence	Article	2023	Nature	491	USA	NIH/NSF/for-profit/non-profit/institution
A practical guide to reflexivity in qualitative research: AMEE Guide No. 149	Consensus	2023	Medical Teacher	490	Colombia	None
2024 Heart Disease and Stroke Statistics: A Report of US and Global Data from the American Heart Association	Reference statistics	2024	Circulation	490	USA	Societies
The molecular and metabolic landscape of iron and ferroptosis in cardiovascular disease	Review	2023	Nature Reviews Cardiology	489	China	NIH/China/institution
Scientific discovery in the age of artificial intelligence	Review	2023	Nature	487	USA	NIH/for-profit/non-profit/institution
2023 ESC Guidelines for the management of cardiovascular disease in patients with diabetes	Consensus	2023	European Heart Journal	486	Germany/Italy	Societies
AASLD Practice Guidance on prevention, diagnosis, and treatment of hepatocellular carcinoma	Consensus	2023	Hepatology	485	USA	Societies
Macrophages in immunoregulation and therapeutics	Review	2023	Signal Transduction and Targeted Therapy	483	China/Sweden	China

© 2025 Ioannidis JPA. *JAMA Health Forum*.

The roles of extracellular vesicles in the immune system	Review	2023	Nature Reviews Immunology	483	Hungary	Hungary
Diagnosis of myelin oligodendrocyte glycoprotein antibody-associated disease: International MOGAD Panel proposed criteria	Consensus	2023	Lancet Neurology	483	USA	Non-profit/institution
Cancer chemotherapy and beyond: Current status, drug candidates, associated risks and progress in targeted therapeutics	Review	2023	Genes and Diseases	480	Spain/India	Spain
Semaglutide in Patients with Heart Failure with Preserved Ejection Fraction and Obesity.	Article	2023	NEJM	477	USA	For-profit/Germany
Autophagy and autophagy-related pathways in cancer	Review	2023	Nature Reviews Molecular Cell Biology	472	USA/UK	NIH/UK/non-profit
Japanese Gastric Cancer Treatment Guidelines 2021 (6th edition)	Consensus	2023	Gastric Cancer	472	Japan	Societies
The evolution of SARS-CoV-2	Review	2023	Nature Reviews Microbiology	470	UK	European
Personalized RNA neoantigen vaccines stimulate T cells in pancreatic cancer	Article	2023	Nature	465	USA	NIH/non-profit/for-profit/institution
Actin cytoskeleton vulnerability to disulfide stress mediates disulfidptosis	Review	2023	Nature Cell Biology	463	USA	NIH/institution
Global, regional, and national burden of low back pain, 1990–2020, its attributable risk factors, and projections to 2050: a	Reference statistics	2023	The Lancet Rheumatology	457	Australia	Multiple international/institution

© 2025 Ioannidis JPA. *JAMA Health Forum*.

systematic analysis of the Global Burden of Disease Study 2021						
Molecular mechanisms of antibiotic resistance revisited	Review	2023	Nature Reviews Microbiology	457	UK	None
Global Cancer Statistics 2022: the trends projection analysis	Review	2023	Chemical Biology Letters	457	USA/India	None
Proksee: In-depth characterization and visualization of bacterial genomes	Resources	2023	Nucleic Acids Research	456	Canada	Canada
Global Initiative for Chronic Obstructive Lung Disease 2023 Report: GOLD Executive Summary	Consensus	2023	European Respiratory Journal	456	Spain	None
American Geriatrics Society 2023 updated AGS Beers Criteria® for potentially inappropriate medication use in older adults	Consensus	2023	Journal of the American Geriatrics Society	444	USA	Societies
Antimicrobial Resistance: A Growing Serious Threat for Global Public Health	Review	2023	Healthcare (Switzerland)	440	Malaysia	None
Perioperative Pembrolizumab for Early-Stage Non-Small-Cell Lung Cancer	Article	2023	New England Journal of Medicine	435	USA	NIH/for-profit
The WHO estimates of excess mortality associated with the COVID-19 pandemic	Article	2023	Nature	435	Switzerland	UN+WHO
Artificial Intelligence and Machine Learning in Clinical Medicine, 2023.	Review	2023	NEJM	432	Norway	None
Dictionary learning for integrative, multimodal and scalable single-cell analysis	Article	2024	Nature Biotechnology	431	USA	NIH/non-profit
Fast and accurate protein structure search with Foldseek	Resources	2024	Nature Biotechnology	430	Germany/S. Korea	Korea/societies/non-profit

© 2025 Ioannidis JPA. *JAMA Health Forum*.

PI3K/AKT/mTOR signaling transduction pathway and targeted therapies in cancer	Review	2023	Molecular Cancer	430	Singapore	Singapore/institution

© 2025 Ioannidis JPA. *JAMA Health Forum*.
