# Supplementary material for: Provenance and Funding of Extremely Cited Biomedical Articles Published Between 2003 and 2024
Source: JAMA Health Forum. 2025 Sep 12;6(9):e253045. doi: 10.1001/jamahealthforum.2025.3045 (PMC12432630; doi:10.1001/jamahealthforum.2025.3045)
Supplement: Supplement 2. — Data Sharing Statement [file jamahealthforum-e253045-s002.pdf]

Data Sharing Statement

Ioannidis. Provenance and Funding of Extremely Cited Biomedical Articles Published Between 2003 and 2024. *JAMA Health Forum*. Published September 12, 2025. doi:10.1001/jamahealthforum.2025.3045

Data
Data available: Yes
Data types: Other (please specify)
Additional Information: Bibliometric data (included in the manuscript and supplements)
How to access data: All data can be found in the manuscript and its supplements
When available: With publication

Supporting Documents
Document types: None

Additional Information
Who can access the data: Anyone (already in the manuscript and supplements)
Types of analyses: Any purpose
Mechanisms of data availability: Provided in the manuscript and its supplements
